# Persistent organic pollutants (POPs) increase rage signaling to promote downstream cardiovascular remodeling

**DOI:** 10.1002/tox.22817

**Published:** 2019-07-16

**Authors:** Jackson B. Coole, Stephanie S. Burr, Amber M. Kay, Jaime A. Singh, Sandeep Kondakala, Eun‐Ju Yang, Barbara L. F. Kaplan, George E. Howell, James A. Stewart

**Affiliations:** ^1^ Department of Biological Sciences, College of Arts and Sciences Mississippi State University Starkville Mississippi; ^2^ Department of BioMolecular Sciences, School of Pharmacy University of Mississippi Oxford Mississippi; ^3^ Virginia Commonwealth University Health Systems Richmond Virginia; ^4^ Department of Basic Sciences, College of Veterinary Medicine Mississippi State University Starkville Mississippi

**Keywords:** AGE‐RAGE signaling, heart, oxidative stress, persistent organic pollutants, type 2 diabetes mellitus

## Abstract

Exposure to environmental contaminants and consumption of a high, saturated fatty diet has been demonstrated to promote precursors for metabolic syndrome (hyperglycemia, hyperinsulinemia, and hypertriglyceridemia). The purpose of this study was to determine if exposure to the most prevalent environmental persistent organic pollutants (POPs) would act as causative agents to promote metabolic syndrome independent of dietary intake. We hypothesized that POPs will activate the advanced glycated end‐product (AGE)‐and receptor for AGE (RAGE) signaling cascade to promote downstream signaling modulators of cardiovascular remodeling and oxidative stress in the heart. At 5‐weeks of age nondiabetic (WT) and diabetic (*ob*/*ob*) mice were exposed POPs mixtures by oral gavage twice a week for 6‐weeks. At the end of 6‐weeks, animals were sacrificed and the hearts were taken for biochemical analysis. Increased activation of the AGE‐RAGE signaling cascade via POPs exposure resulted in elevated levels of fibroblast differentiation (α‐smooth muscle actin) and RAGE expression indicated maladaptive cardiac remodeling. Conversely, the observed decreased superoxide dismutase‐1 and ‐2 (SOD‐1 and SOD‐2) expression may exacerbate the adverse changes occurring as a result of POPs treatment to reduce innate cardioprotective mechanisms. In comparison, ventricular collagen levels were decreased in mice exposed to POPs. In conclusion, exposure to organic environmental pollutants may intensify oxidative and inflammatory stressors to overwhelm protective mechanisms allowing for adverse cardiac remodeling.

## 1 INTRODUCTION

Recently, there has been an increasing interest in investigating the effects of persistent organic pollutants (POPs) exposure and its association with the prevalence of type 2 diabetes mellitus (T2DM) and the metabolic syndrome. POPs are organic compounds that resist photolytic, biological, and chemical degradation.^1^ These compounds are hydrophobic and lipophilic chemicals that are stored in the lipids of organisms due to slow degradation of these compounds.^2^ This phenomenon will lead to bioaccumulation and eventually transport of POPs upwards in the food chain.^2^ Exposure to POPs occurs primarily through the consumption of food, especially meat, fish, and dairy products, containing environmental pollutants. Interestingly, POPs also have the capability to travel long‐range distances via their natural tendency to enter the gas phase under environmental temperatures.^3^ Therefore, POPs found in food do not always come from the pesticides used in the vicinity of industries located near food‐producing farms.^3^

Epidemiological studies indicate exposure to certain organochlorine (OC) pesticides is positively associated with the development of insulin resistance, diabetes, and the metabolic syndrome.^4^, ^5^ Animal studies have revealed that exposure to POPs in the presence of fatty fish oil led to the development of insulin resistance, abdominal obesity, and hepatosteatosis.^6^, ^7^ The analysis of data from the National Health and Nutrition Examination Survey (NHANES) from 1999 to 2002 indicated that diabetic prevalence in individuals was strongly positively associated with concentrations of POPs serum concentrations, with OC pesticides being most strongly and consistently associated with the metabolic syndrome.^8^, ^9^ Though the underlying mechanism(s) of action governing the effects of POPs exposure remains poorly understood, these studies have shown that POPs, especially the OC pesticides or their metabolites as well as polychlorinated biphenyls (PCBs), are positively correlated with the susceptibility to and onset of T2DM.^7^, ^8^, ^9^, ^10^ Additionally, recent epidemiological studies have indicated that elevated levels of POPs are positively associated with alterations in left ventricle geometry and function in the elderly (PMID 23743808 and PMID 23562393).^11^, ^12^ Thus, the possibility arises that elevated levels of POPs may alter cardiac remodeling and function.

Increased circulating levels of glucose, as observed in diabetic patients, will react nonenzymatically with amino groups at the carbonyl ends of proteins to form reversible Schiff bases and then Amadori compounds through a process known as the Maillard reaction.^13^ The resulting product is an irreversibly crosslinked derivative termed advanced glycation end products (AGEs).^14^ Other pathways for AGE production include autoxidation of glucose from an increase in oxidative stress, and the conversion of glucose to sorbitol and then to fructose, known as the polyol pathway.^15^, ^16^, ^17^ Evidence also suggests that AGEs form during natural aging because of exposure of long‐lived proteins to euglycemic levels.^14^, ^18^, ^19^, ^20^, ^21^ Due to the diverse pathways leading to AGE genesis, AGE chemical structures tend to vary; however, accumulation of assorted AGEs will alter cellular and intracellular structures as well as function through activation of the multi‐ligand receptor for AGEs or RAGEs. RAGEs are transmembrane cell surface receptors whose cytoplasmic domain is key for downstream signaling.^14^ The AGE‐RAGE signaling pathway can trigger multiple cellular signaling pathways in which various downstream signaling pathways, such as Ras‐extracellular signal regulated protein kinase (ERK1/2), Jak/Stat, and the transcription regulator nuclear factor‐κB (NF‐κB) are involved in the generation of reactive oxygen species (ROS).^22^, ^23^, ^24^

In a previous a study by Mulligan et al, the experimental design and POPs mixture exposure were reported to successfully promote nonalcoholic fatty liver disease (NAFLD) hepatic steatosis in *ob*/*ob* mice.^24^, ^25^ The purpose of this study was to determine if the POPs exposure in the same animals would act as causative agents to promote metabolic syndrome outcomes in the heart independent of dietary intake. We hypothesized that the AGE‐RAGE signaling cascade will be activated by POPs exposure to stimulate downstream signaling modulators to promote cardiovascular remodeling. Given the critical role of activation of the AGE‐RAGE axis in the pathogenesis of T2DM and cardiac remodeling, these findings demonstrate that exposure to POPs promotes cardiometabolic risk and cardiac remodeling, due as least in part to POPs‐induced activation of the AGE‐RAGE axis. In states of metabolic dysfunction such as obesity and diabetes, ROS levels become exceedingly high, and when compounded with POPs exposure, innate antioxidant defense mechanisms become overwhelmed with increased levels of ROS from the activation of RAGE/NF‐κB signaling.^24^, ^26^ The following results demonstrate a potential role for AGE‐RAGE signaling in the POPs‐mediated remodeling.

## 2 MATERIALS AND METHODS

### 2.1 Animal care and use

Four‐week old, male leptin‐deficient *ob*/*ob* (*Lep−*/*−*) and male wild type (WT, C57BL/6J) mice were acquired from Jackson Laboratories. Animals were individually housed in an AAALAC‐approved animal facility. Food and water were provided ad libitum unless otherwise indicated. The Mississippi State University Animal Care and Use Committee approved all animal usage protocols.

### 2.2 Animal model and POPs mixture

In a previous a study by Mulligan et al, the experimental design and POPs mixture were reported to successfully promote NAFLD hepatic steatosis in *ob*/*ob* mice.^24^ Briefly, four‐week old male WT and *ob*/*ob* mice were randomly divided into treatment groups with 10 animals per group. Treatment groups are as follows: (a) WT, (b) *ob*/*ob*, (c) WT POPs, and (d) *ob*/*ob* POPs. The POPs mixture contained the five most prevalent OC pesticide or their metabolites as well as the five most prevalent PCBs present in contaminated salmon.^7^, ^24^ The following chemicals were contained within the POPs mixture: pp‐dichlorodiphenyldichloroethylene (DDE), pp‐dichlorodiphenyldichloroethane (DDD), hexachlorobenzene, dieldrin, transnonachlor, PCB‐153, PCB‐138, PCB‐118, PCB‐77, and PCB‐126, Table 1. A twice weekly oral gavage method was used to administer POPs treatments for 7 weeks at which time the animals were sacrificed. It should be noted that POPs were delivered in a corn oil vehicle (1 mL/kg) to mimic oil dissolved POPs, as would occur in contaminated food. The weekly intake of POPs yielded equivalent concentrations of POPs as that in previously published studies based on contaminated salmon levels and therefore reflects an environmentally relevant exposure via dietary intake.^7^, ^10^, ^24^ Serum and adipose concentrations of POPs were not measured, as they were used as experimental endpoints for alternative studies previously reported.^24^ Prior to sacrificing, mice were weighed and fasting glucose levels were recorded by glucometer. Hearts were removed, weighed, and sectioned for paraformaldehyde fixation and freezing at −80°C for biochemical assays. Serum was collected to measure fasting insulin levels which were previously published by Mulligan et al.^24^

**Table 1:** Administered persistent organic pollutants (POPs). Persistent organic pollutants (POPs) and their administered concentrations were recorded in the table above. POPs daily intake was based on an average of 4 g/day food consumption^24^

POP compound	ng/g diet	Daily intake (ng)	Bi‐weekly intake (ng)
pp‐DDE	7.16	28.64	100.24
pp‐DDD	3.57	14.28	49.98
Hexachlorobenzene	1.61	6.44	22.54
Dieldrin	1.58	6.32	22.12
Transnonachlor	1.29	5.16	18.06
PCB‐153	2.35	9.4	32.90
PCB‐138	2.33	9.32	32.62
PCB‐118	1.31435	5.2574	18.40
PCB‐77	0.0237	0.0948	0.33
PCB‐126	0.00778	0.03112	0.11

### 2.3 Protein isolation

After hearts were harvested, proteins were isolated as follows. 500 μL of modified Hunter's Buffer, containing 1% Triton X‐100, 75 mM NaCl, 5 mM Tris (pH 7.4), 0.5 mM orthovanadate, 0.5 mM EDTA, 0.5 mM EGTA, 0.25% NP‐40 and protease inhibitors, was added to 50 mg of heart tissue. On ice, tissue was minced and then sonicated for 5 second bursts until it was disrupted. Lysates were centrifuged for 15 minutes at 20 000*g* at 4°C. The supernatant was transferred to new 1.5 mL centrifuge tubes and stored at −80°C. Protein concentration for each sample was determined using the bicinchoninic acid (BCA) assay (Pierce Biotechnology) according to manufacturer's instructions.

### 2.4 Western blot protein analysis

Equal amounts of tissue lysate (25 μg) were separated on a 10% sodium dodecyl sulfate (SDS)‐polyacrylamide gel (BioRad Laboratories) for approximately 2 to 3 hours at 100 V at 4°C. Proteins were electrophoretically transferred to nitrocellulose membranes overnight at 100 mA at 4°C. Membranes were stained with Coomassie Brilliant Blue Dye (Fisher Scientific) to verify even transfer. Membranes were blocked in either 5% powdered milk or 5% BSA in Tris buffered saline with 0.1% Tween 20 and then incubated with the following antibodies: cardiac fibroblast phenotype marker α‐smooth muscle actin (α‐SMA, Sigma‐Aldrich), ECM regulators (RAGE, Abcam) and signaling proteins (phospho and total NF‐kB, Santa Cruz Biotechnologies) while GAPDH (Santa Cruz Biotechnologies) was used as a loading control. Blots were developed using Pierce enhanced chemiluminescence (ECL) solution (Pierce Biotechnology), exposed to X‐ray film, and immunoreactive bands were quantified using NIH Image J. Measured densitometric values were normalized to their respective loading controls (ie, GAPDH, total NF‐κB) and then normalized to WT‐Sham. Changes in protein expression are described as being a fold increase or fold decrease of Sham. Representative blots shown in Results section may include membranes that have been probed, stripped, and reprobed multiple times. For these images, it will be denoted in the figure legends.

### 2.5 Histological analysis

Morphological evaluation of hematoxylin and eosin (H&E) stained slides was performed on hearts from WT, *ob*/*ob*, WT + POPs and *ob*/*ob* + POPs mice for neutrophil infiltration into the myocardium. Briefly, hearts were fixed in 10% buffered formalin and embedded in paraffin blocks. Blocks were sectioned at 5‐μm thickness and stained with H&E, and estimation of neutrophils was obtained by using bright field microscopy.

### 2.6 Collagen content

Myocardial collagen concentration and solubility was determined as described.^27^, ^28^ In brief, 50 mg of cardiac tissue was mixed with 3 mL of 200 μg/mL pepsin in 0.5 M acetic acid and incubated at 37°C with gentle agitation. Following 2 hours of digestion, samples were centrifuged at 20 000*g* for 20 minutes at 4°C and 1 mL of supernatant was removed to be frozen at −80°C until Sircol collagen analysis. The pellet was resuspended in the remaining supernatant to be incubated at 37°C with gentle agitation for 24 hours. Following 24 hours of digestion, samples were centrifuged at 20 000*g* for 20 minutes at 4°C and another 1 mL aliquot of supernatant was removed to be frozen at −80°C until Sircol analysis. The pellet was then resuspended in the remaining supernatant to be incubated at 110°C in a 6 M HCl solution for 18 hours. Following 18 hours of 6 M HCl digestion, samples were centrifuged at 20 000*g* for 20 minutes at 4°C and 1 mL of supernatant was discarded. The remaining pellet was resuspended in deionized water and frozen at −80°C until Sircol analysis. Sircol collagen assay (Biocolor, Ltd., Ireland) was performed according to manufacturer's instructions. The conditions for pepsin digestion were chosen, so that significant solubilization of the unmodified or soluble collagen was identified at 2 hours and 24 hours, which maximum solubilization (>98%) was observed.^27^, ^28^ Insoluble collagen content was determined by measuring collagen concentration after 18 hours acid hydrolysis using the procedure of Stegemann and Stalder.^27^ Collagen content was expressed as μg of collagen per mg of wet weight of heart tissue.

Morphological evaluation of picric acid‐sirius red stained collagen was performed on hearts from WT, *ob*/*ob*, WT + POPs and *ob*/*ob* + POPs mice as previously described.^29^, ^30^ Briefly, hearts were fixed in 10% buffered formalin and embedded in paraffin blocks. Blocks were sectioned at 5‐μm thickness and stained with Picric acid‐sirius red F3BA. Estimates of the fractions of collagen fibrils were obtained by using polarized light. Due to the birefringent quality of the stain, collagen refracted a distinct color based upon the size of the collagen fibrils: red and yellow (thick filaments) and green (thin filaments). Quantitative analysis is accomplished by light microscopy with a video‐based image‐analyzer system. Color thresholds were set for biphasic analysis to capture and generate a percent collagen content per 20× field within the specified RGB wavelength ranges separate from the background: Phase 1 represents collagen capture Red (0‐40) Green (0‐80) Blue (0‐255) and Phase 2 represents background capture: Red (20‐255) Green (40‐255) Blue (35‐255). Results are presented as the mean ± SEM values computed from the average of n = 20‐30 individual measurements obtained from each heart analyzed. Cardiac vasculature, epicardium, and endocardium were avoided due to high levels of collagen content which does not accurately reflect myocardial interstitial collagen.^29^, ^30^

### 2.7 Statistical analysis

For morphometric data analysis, western blot protein analysis, and collagen content analysis, a one‐way ANOVA performed with Tukey's post hoc analysis to examine differences between WT, WT‐POPs, *ob*/*ob*, and *ob*/*ob*‐POPs mice. Statistical analysis was performed using GraphPad Prism 5 software. Statistical differences are defined as data having *P* < .05. Error bars represent ± SE of the mean (SEM).

## 3 RESULTS

### 3.1 Physiological and biochemical markers of obesity and T2DM resulting from POPs exposure and genotype

The data presented herein were gathered using heart tissue samples from identical animals as those used by Mulligan et al.^24^ Briefly, *ob*/*ob* POPs and *ob*/*ob* mice body weights were significantly heavier than that of their WT littermates indicating obesity.^24^ Additionally, *ob*/*ob* POPs and *ob*/*ob* mice had higher fasting blood glucose and insulin levels.^24^ When hearts were examined for changes in morphology, such as hypertrophy or dilatation, there were no differences in heart weight, Figure 1A. Significant differences were observed in the ventricular to body weight ratios, Figure 1B. Diabetic *ob*/*ob* mice had significantly smaller ventricular to body weight ratios than their lean WT littermates. These findings indicate a larger body weight or obese phenotype observed in the *ob*/*ob* mice. Most notably Mulligan et al found significant increases in fasting triglyceride and total cholesterol in *ob*/*ob* mice compared to WT; however, no differences were observed between untreated and POPs treated animals.^24^ There was also marked triglyceride accumulation in the liver indicating increased hepatic steatosis.^24^ Yet, these changes were significantly higher in *ob*/*ob* POPs than *ob*/*ob* groups. From this prior study, it was found that POPs treatment significantly increased chemokine (C‐C motif) ligand 2 (CCL2 or monocyte chemoattractant protein 1, MCP1) proinflammatory cytokine expression by approximately 10‐fold in the white adipose tissue of *ob*/*ob* animals indicating an additional inflammatory response.^24^

**Figure 1:**
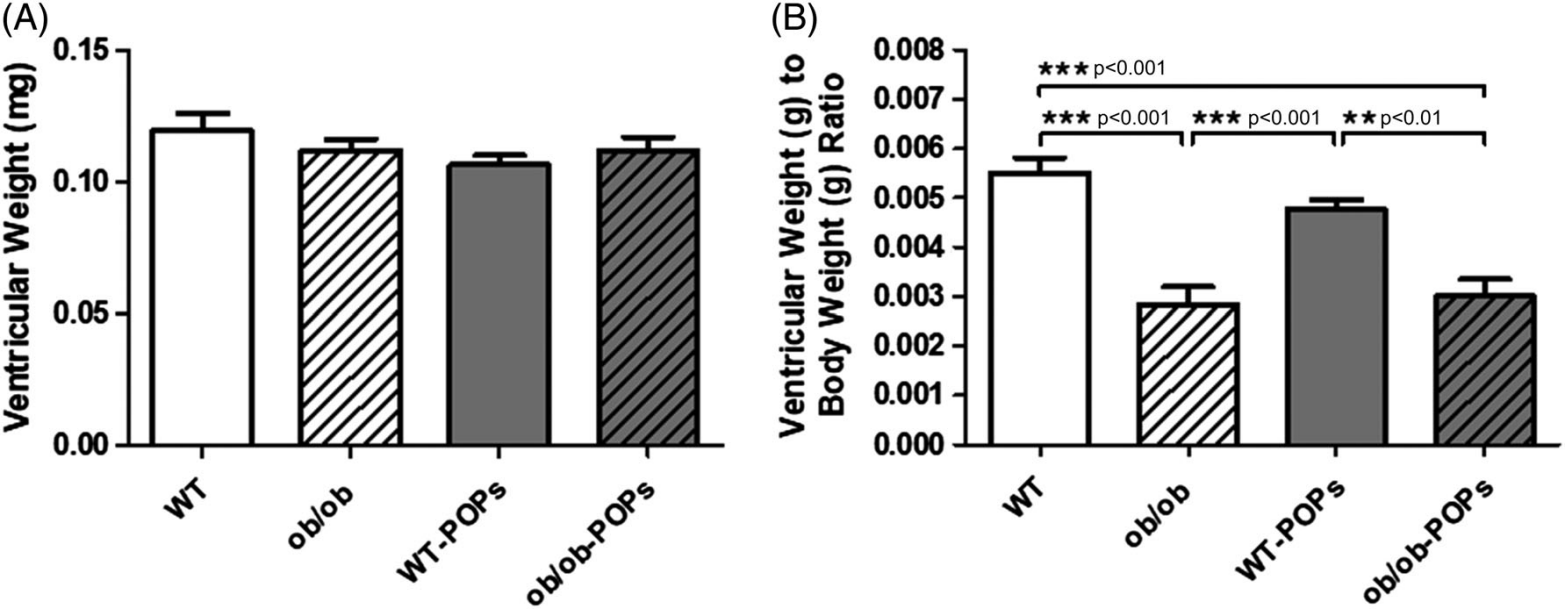
A, Ventricular weight. Hearts were examined for changes in morphology, such as hypertrophy, there was no differences in heart weight between wildtype (WT) and diabetic (*ob*/*ob*) hearts. POPs treatment did not alter heart weight. n = 10‐11 hearts per group. B, Ventricular weight to body weight ratio. Significant differences were observed in the ventricular weight to body weight ratios in diabetic *ob*/*ob* mice. There was a significant decrease in ratios in diabetic as compared to their lean wildtype (WT) littermates. This finding indicates a larger body weight or obese phenotype observed in the *ob*/*ob* mice that was unaffected by persistent organic pollutant (POPs) exposure. n = 10‐11 hearts per group. A one‐way ANOVA with Tukey's post hoc analysis was performed to examine differences between WT, WT‐POPs, *ob*/*ob*, and *ob*/*ob*‐POPs mice. Statistical differences are denoted on the graph. Error bars represent ± SE of the mean (SEM)

### 3.2 Signaling protein analysis

#### 3.2.1 RAGE

**Figure 2:**
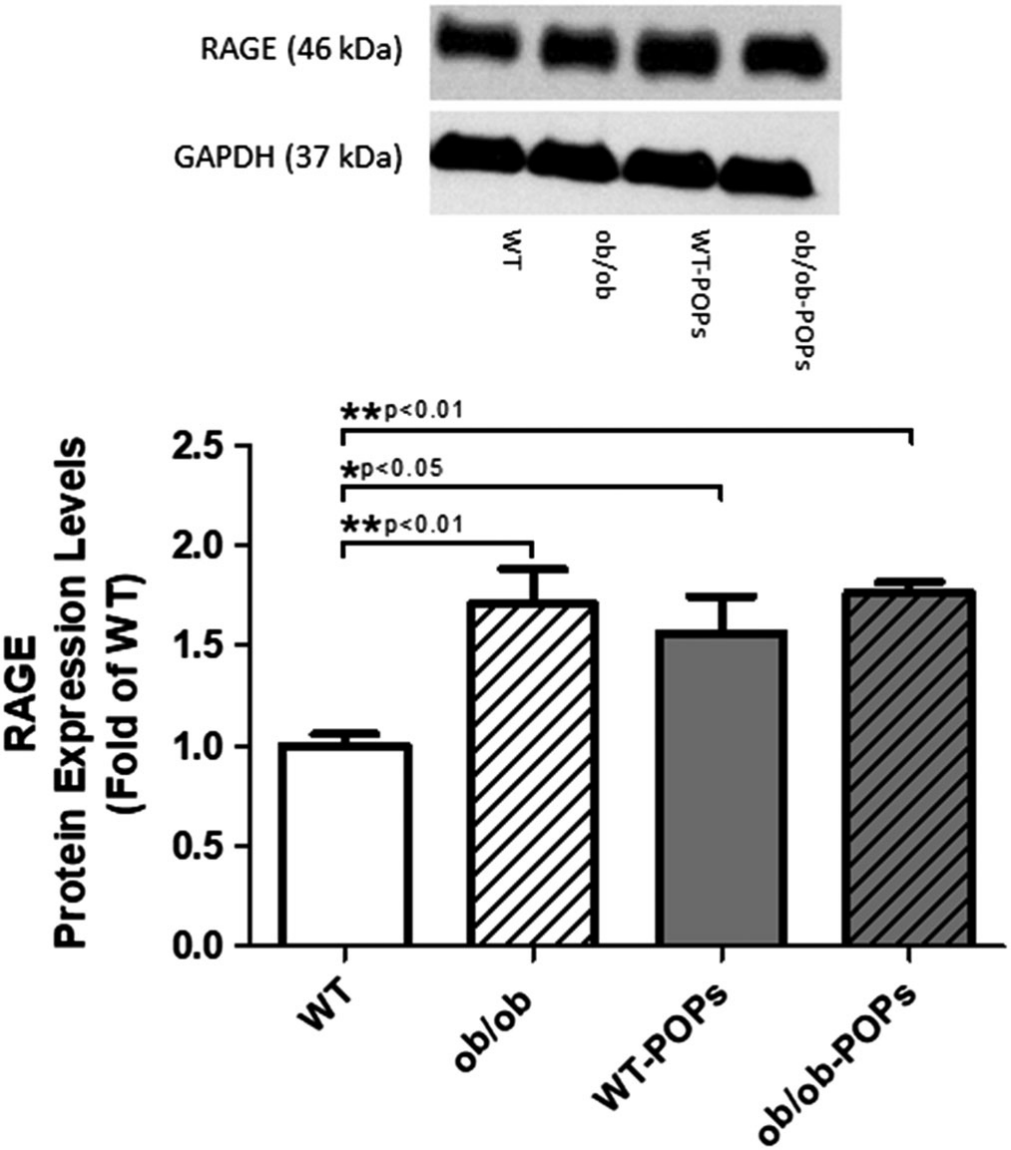
RAGE protein expression. RAGE protein expression was significantly increased in WT‐POPs, *ob*/*ob*, and *ob*/*ob*‐POPs treated hearts as compared to WT. n = 10‐11 hearts per group. Representative blots shown may include membranes that have been probed, stripped, and reprobed multiple times. A one‐way ANOVA with Tukey's post hoc analysis was performed to examine differences between WT, WT‐POPs, *ob*/*ob*, and *ob*/*ob*‐POPs mice. Statistical differences are denoted on the graph. Error bars represent ± SE of the mean (SEM)

RAGE expression was significantly increased in WT‐POPs, *ob*/*ob*, and *ob*/*ob*‐POPs treated hearts as compared to WT hearts, Figure 2. These findings correlated with previous studies in which RAGE expression was reported to be significantly elevated in tissues of the of diabetic *ob*/*ob* mice.^31^ These observations are the first of its kind to implicate organic pollutants as potential promoters of RAGE protein expression. Finding significantly elevated RAGE levels in WT‐POPs hearts suggests a possible role for POPs in activating the AGE‐RAGE signaling cascade independent of hyperglycemia. Further studies will need to be conducted to determine the potential mechanisms responsible for increased RAGE expression in POPs treated animals in order to understand the maladaptive responses resulting from POPs‐mediated increased AGE‐RAGE signaling.

#### 3.2.2 α‐Smooth muscle actin (SMA)

**Figure 3:**
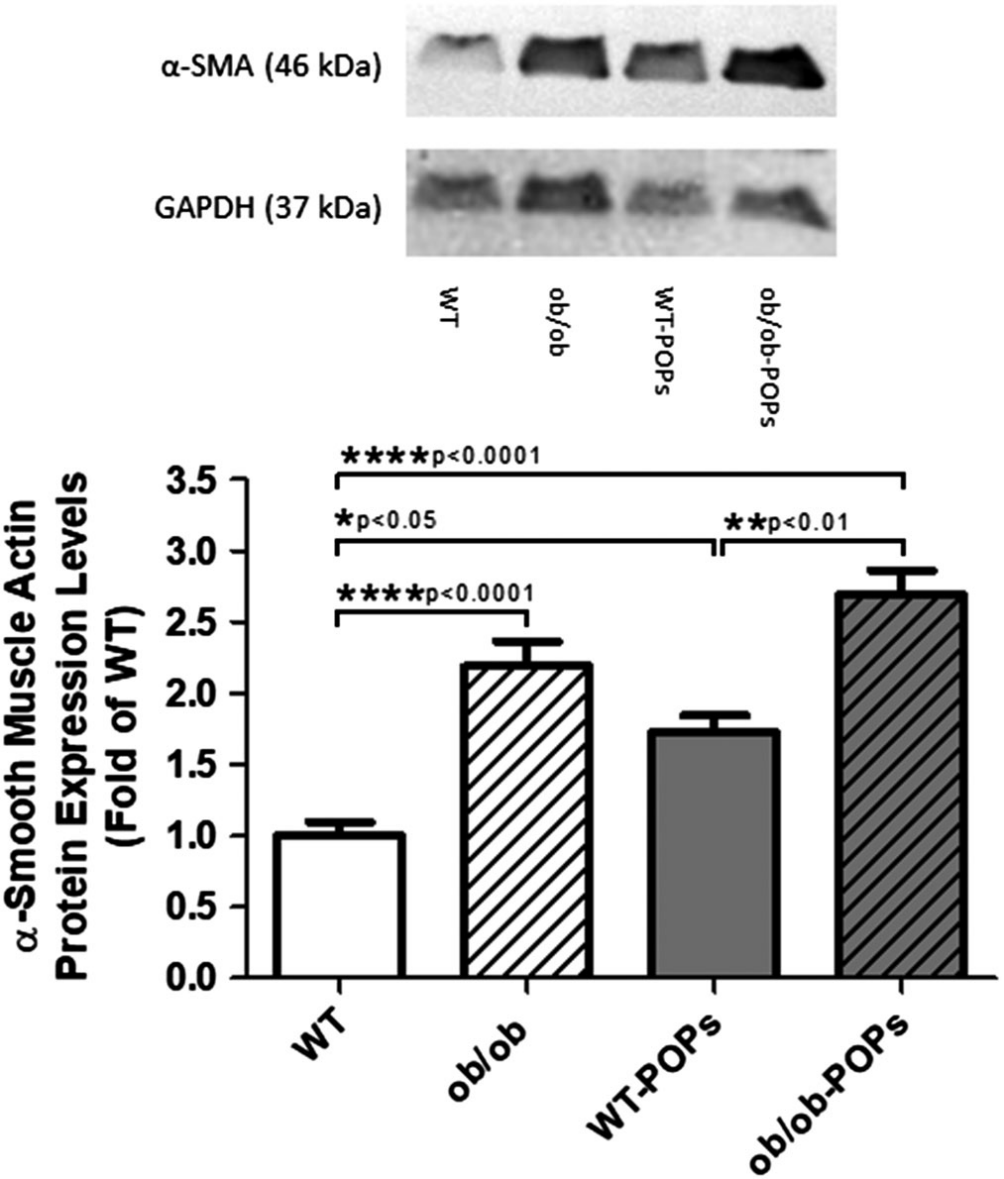
α‐Smooth muscle actin (SMA) protein expression. α‐Smooth muscle actin (SMA) protein expression was found to be significantly elevated in the *ob*/*ob*, WT‐POPs, and *ob*/*ob*‐POPs animals compared to WT hearts. n = 10‐11 hearts per group. Representative blots shown may include membranes that have been probed, stripped, and reprobed multiple times. A one‐way ANOVA with Tukey's post hoc analysis was performed to examine differences between WT, WT‐POPs, *ob*/*ob*, and *ob*/*ob*‐POPs mice. Statistical differences are denoted on the graph. Error bars represent ± SE of the mean (SEM)

Previously published studies by our laboratory demonstrated a role for AGE‐RAGE signaling in fibroblast stress responses.^30^ Of note, increased α‐smooth muscle actin (SMA) protein expression has been shown to be present in diabetic fibroblast undergoing fibroblast‐to‐myofibroblast differentiation in response to elevated AGE‐RAGE signaling.^30^ Western blot analysis was used to determine changes in fibroblast differentiation in the ventricular samples. α‐SMA protein expression was found to be elevated in *ob*/*ob* and in POPs treated WT and *ob*/*ob* animals compared to hearts of WT mice, Figure 3. Interestingly, WT‐POPs and *ob*/*ob* α‐SMA levels were not significantly different. Thus, indicating a potential rise in the stressed fibroblast phenotype within the hearts of WT‐POPs mice independent of circulating glucose levels. These changes may be attributed to increases in AGE‐RAGE signaling.

#### 3.2.3 Phospho‐NF‐κB

**Figure 4:**
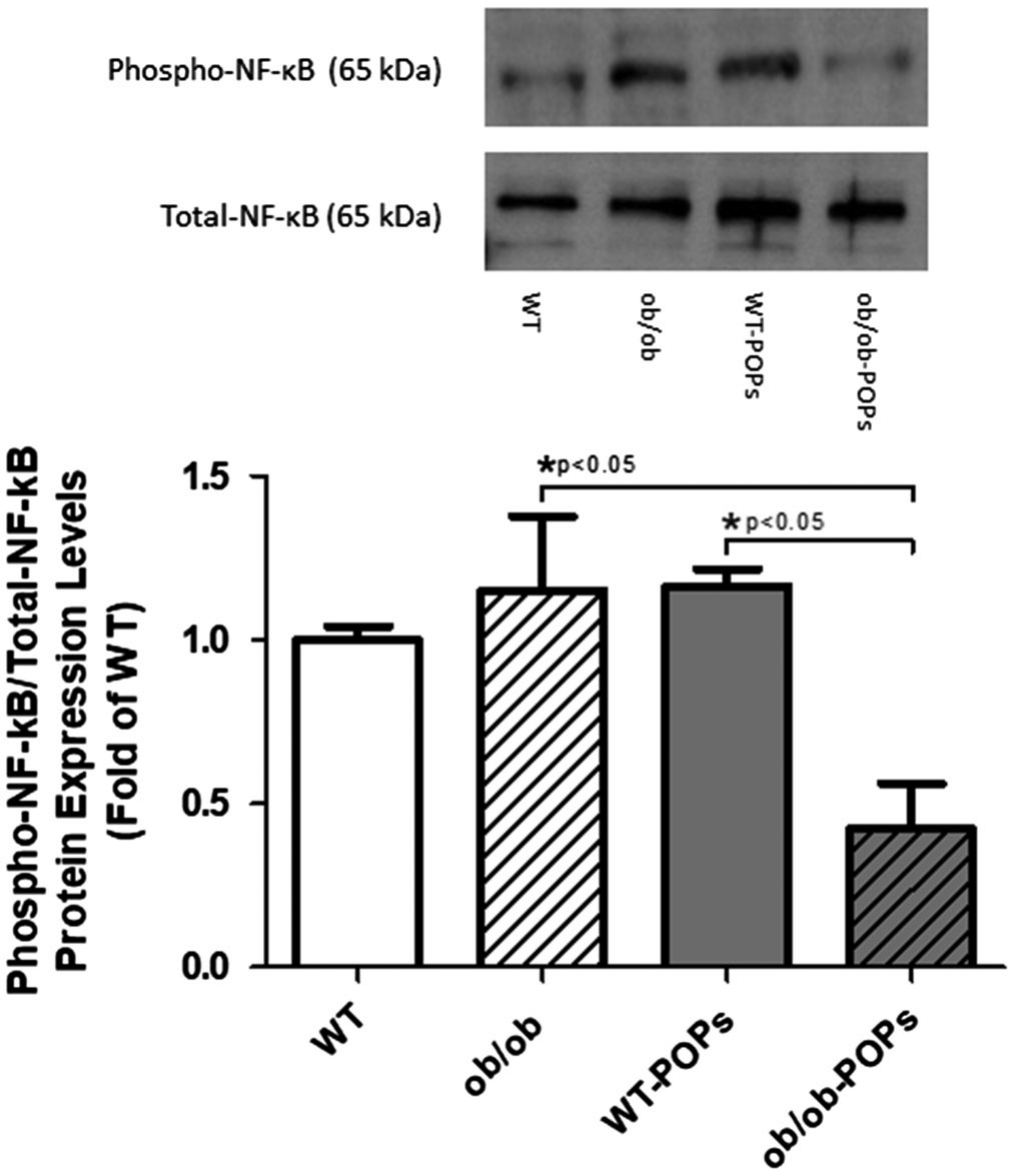
Phospho‐NF‐κB/total‐NF‐κB protein expression. Phospho‐NF‐κB (active NF‐κB) as shown by western blot analysis was found to be significantly decreased in *ob*/*ob*‐POPs hearts as compared to *ob*/*ob* and WT‐POPs hearts. n = 10‐11 hearts per group. Representative blots shown may include membranes that have been probed, stripped, and reprobed multiple times. A one‐way ANOVA with Tukey's post hoc analysis was performed to examine differences between WT, WT‐POPs, *ob*/*ob*, and *ob*/*ob*‐POPs mice. Statistical differences are denoted on the graph. Error bars represent ± SE of the mean (SEM)

In addition to stimulating fibroblast differentiation, AGE‐RAGE cascade activation will also alter a number of proinflammatory modulators, such as NF‐κB.^32^ Western blot analysis showed slightly elevated levels of phospho‐NF‐κB, the active form of NF‐κB, demonstrated to increase levels of oxidative stress, in *ob*/*ob* and WT‐POPs animals as compared to WT mice; however, these changes were not significant, Figure 4. Furthermore, POPs exposure in *ob*/*ob* hearts significantly decreased NF‐κB phosphorylation levels below those measured in *ob*/*ob* and WT‐POPs mouse ventricular samples.

#### 3.2.4 SOD‐1 and SOD‐2 levels

**Figure 5:**
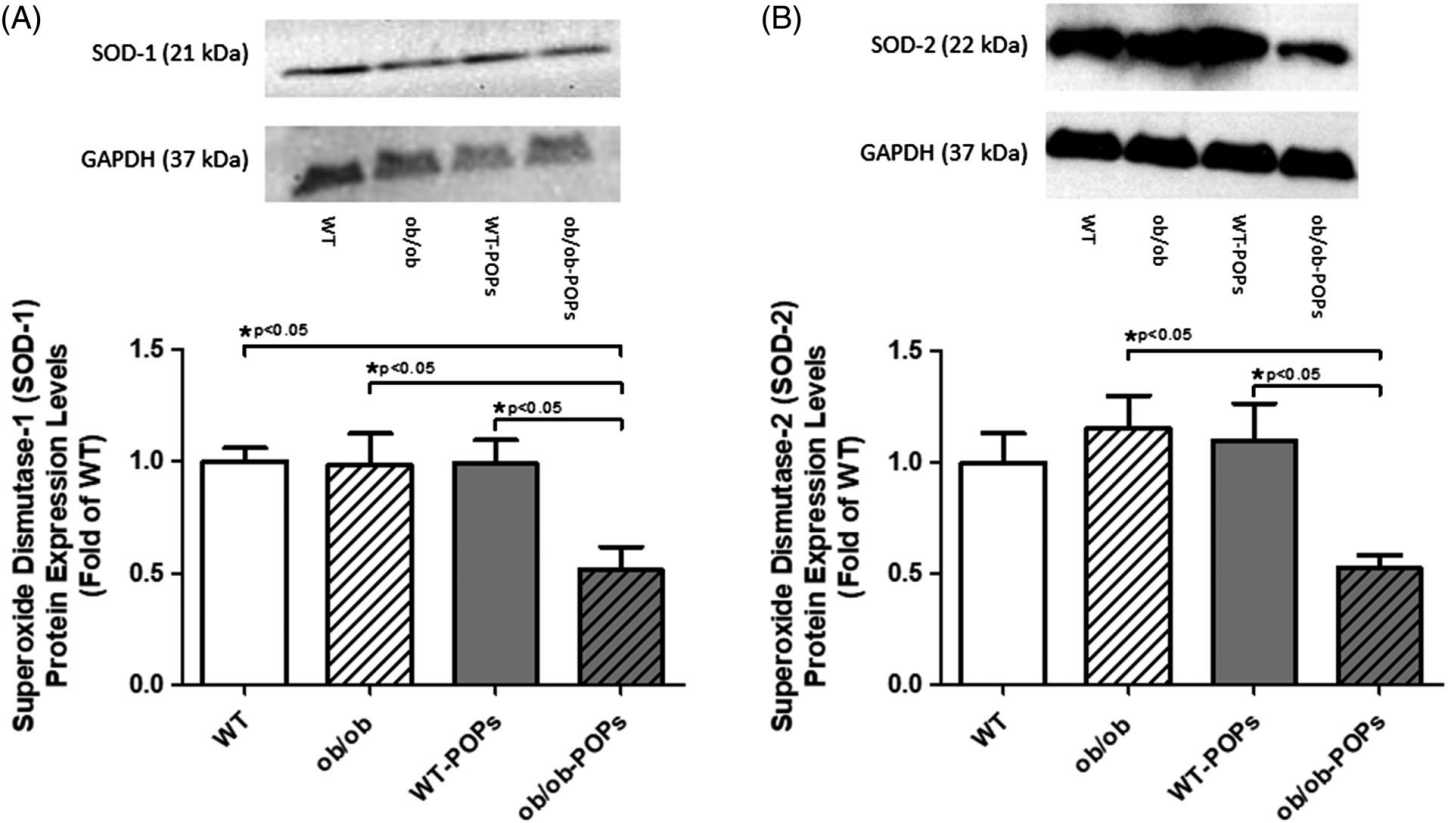
A, SOD‐1 protein expression. Superoxide dismutase‐1 (SOD‐1) was significantly decreased *ob*/*ob*‐POPs compared to WT, *ob*/ob, and WT‐POPs mice hearts. n = 10‐11 hearts per group. B, SOD‐2 protein expression. Superoxide dismutase‐2 (SOD‐2) was significantly decreased *ob*/*ob*‐POPs compared to *ob*/ob and WT‐POPs mice hearts n = 10‐11 hearts per group. Representative blots shown in may include membranes that have been probed, stripped, and reprobed multiple times. A one‐way ANOVA with Tukey's post hoc analysis was performed to examine differences between WT, WT‐POPs, *ob*/*ob*, and *ob*/*ob*‐POPs mice. Statistical differences are denoted on the graph. Error bars represent ± SE of the mean (SEM)

Superoxide dismutase‐1 and ‐2 (SOD‐1 and SOD‐2) are generally ubiquitously expressed, innate antioxidants used to neutralize superoxide radicals in the body.^19^ In this study SOD‐1 and ‐2 protein expression levels were measured to determine how efficiently POPs treated animals were able to compensate for increased oxidative stressors. POPs exposure in *ob*/*ob* mice significantly decreased both SOD‐1 and SOD‐2, Figure 5A,B, respectively, as compared to WT, *ob*/*ob*, WT‐POPs groups. We predict that decreased SOD‐1 and ‐2 levels in *ob*/ob‐POPs mice correlated with their inability to compensate with rising levels of oxidative stressors in the hearts. These data may suggest the *ob/ob*‐POPs mice have lost the ability to neutralize superoxide radicals.

#### 3.2.5 Histological analysis

**Figure 6:**
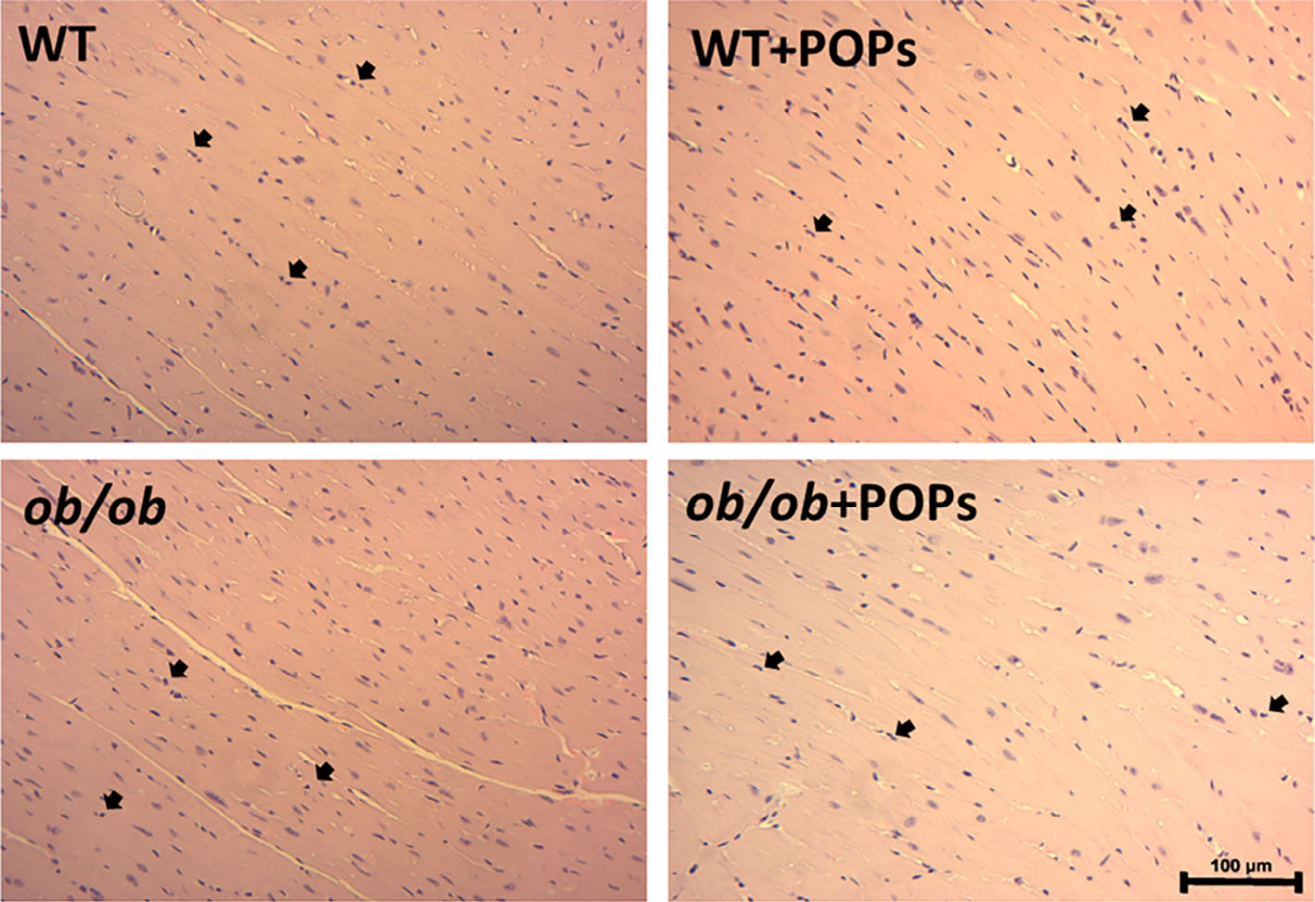
Histological evaluation. Morphological evaluation of hematoxylin and eosin (H&E) stained slides was performed on hearts from WT, *ob*/ob, WT + POPs and *ob*/*ob* + POPs mice for neutrophil infiltration into the myocardium. Estimation of neutrophils was obtained by using ×200 bright field microscopy. Neutrophils were denoted by arrowheads. There were no differences in the number of neutrophils

Morphological evaluation of H&E was performed on hearts from WT, *ob*/*ob*, WT + POPs and *ob*/*ob* + POPs mice. Neutrophil infiltration into the myocardium was estimated. There were no significant differences in neutrophil population across the study groups (Figure 6).

#### 3.2.6 Collagen content

Collagen content was measured to determine downstream effectors of the AGE‐RAGE signaling cascade. Ventricular fibrosis has been demonstrated to be a significant outcome of increased RAGE activation.^30^ POPs exposure in both WT and *ob*/*ob* mouse hearts had significantly decreased soluble and insoluble collagen levels, Figure 1A,B. As a result, soluble‐insoluble ratios were also decreased, Figure 1C. Collectively, these results can indicate low collagen production, increased collagen degradation by matrix metalloproteases (MMPs), as well as decreased collagen crosslinking, which can impact cardiac performance. These findings were also observed in picric acid‐sirius red stained ventricular cross‐sections; however, the percent of stained collagen was significantly decreased in *ob*/*ob*‐POPs hearts (Figure 7).

**Figures 1:**
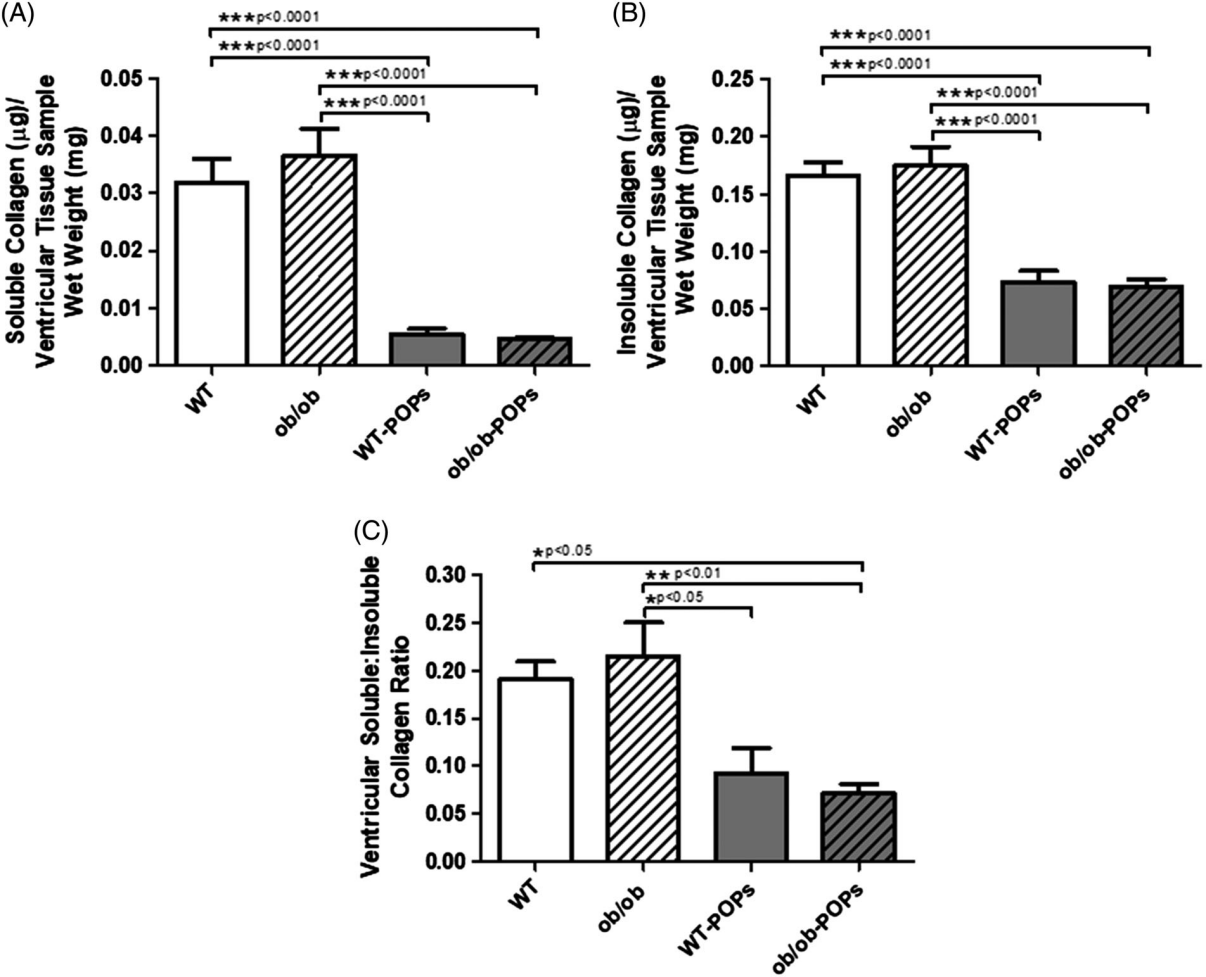
A, Soluble collagen. Soluble collagen was significantly decreased in WT‐POPs and *ob*/*ob*‐POPs compared to WT and *ob*/ob mice hearts. n = 10‐11 hearts per group. B, Insoluble collagen. Insoluble collagen was significantly decreased in WT‐POPs and *ob*/*ob*‐POPs compared to WT and *ob*/ob mice hearts. n = 10‐11 hearts per group. C, Soluble:Insoluble ratio. Soluble:Insoluble collagen was significantly decreased in WT‐POPs and *ob*/*ob*‐POPs compared to WT and *ob*/ob mice hearts. n = 10‐11 hearts per group. A one‐way ANOVA with Tukey's post hoc analysis was performed to examine differences between WT, WT‐POPs, *ob*/*ob*, and *ob*/*ob*‐POPs mice. Statistical differences are denoted on the graph. Error bars represent ± SE of the mean (SEM)

**Figure 7:**
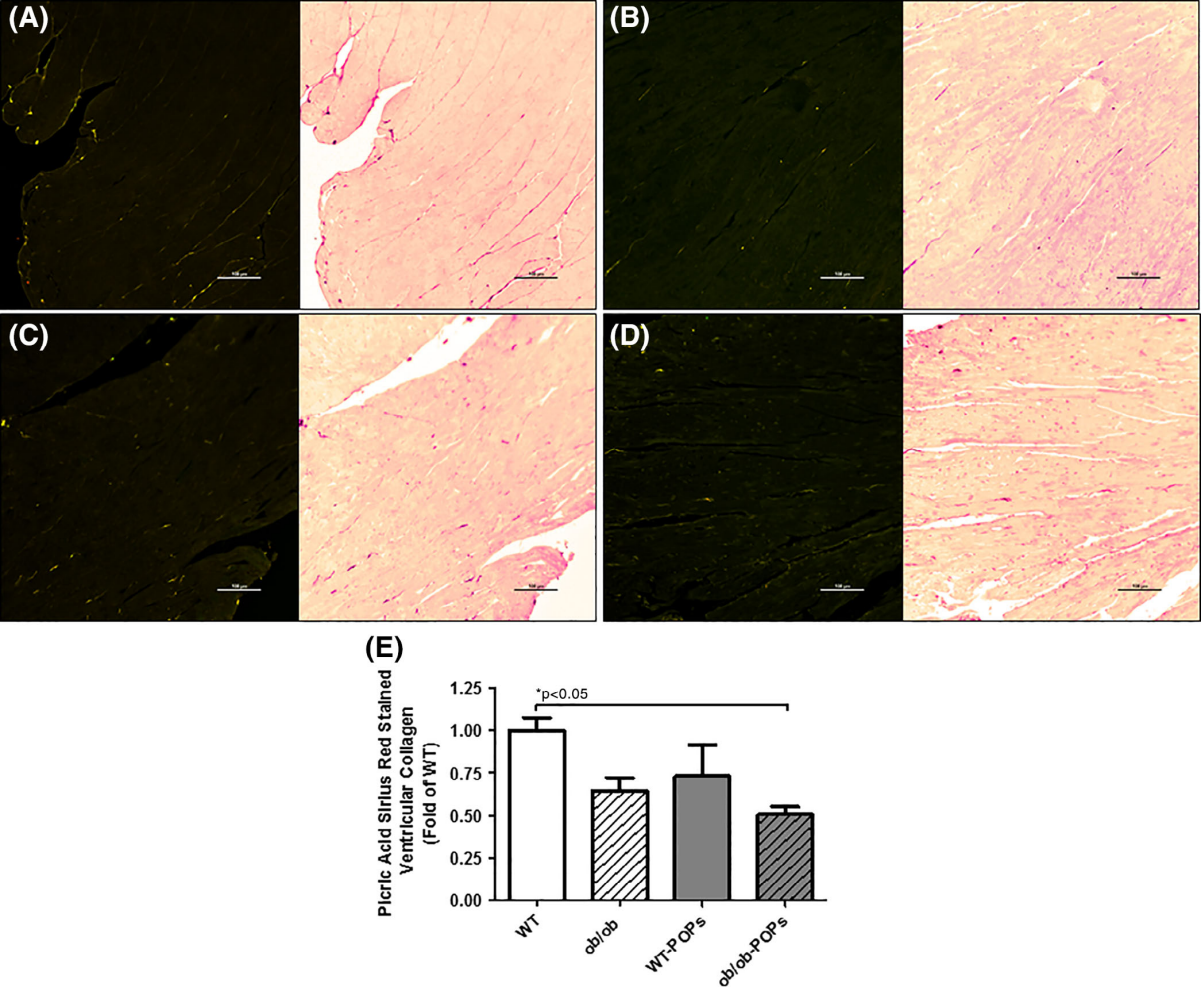
A‐E, Picric acid‐sirius red stained ventricles. Stained collagen was visualized using polarized light (left panel) as well as brightfield (right panel) microscopy. A, WT representative images. B, *ob*/*ob* representative images. C, WT‐POPs representative images. D, *ob*/*ob*‐POPs representative images. E, Stained collagen was significantly decreased in *ob*/*ob*‐POPs compared to WT hearts. n = 5‐6 hearts per group. A one‐way ANOVA with Tukey's post hoc analysis was performed to examine differences between WT, WT‐POPs, *ob*/*ob*, and *ob*/*ob*‐POPs mice. Statistical differences are denoted on the graph. Error bars represent ± SE of the mean (SEM). Scale bars equals 100 μm

## 4 DISCUSSION

From previous studies, increased POPs exposure exacerbated hepatic steatosis and adipocyte dysfunction resulting in elevated production of proinflammatory and oxidative stressors.^24^ These changes in the inflammatory profile have been demonstrated to exacerbate type 2 diabetes mellitus (T2DM); however, the mechanism regulating this process is unknown.^10^Therefore, our present study aimed to determine if POPs exposure would act as a causative agent to promote metabolic syndrome outcomes in the heart independent of dietary intake. We hypothesized that activation of the AGE‐RAGE signaling cascade by POPs exposure would stimulate downstream signaling modulators to promote cardiovascular remodeling. Given the critical role of the AGE‐RAGE axis in the pathogenesis of T2DM and cardiac remodeling, these findings demonstrate that exposure to POPs increased cardiometabolic risk and cardiac remodeling. Thus, our current data represent a plausible mechanism through which POPs exposure may promote alterations in left ventricular geometry and function as demonstrated in recent epidemiological studies in the elderly.^11^, ^12^ It should be noted that while the mean serum levels of some of the POPs, such as the organochlorine pesticide metabolites and some of the PCBs, are declining in U.S. population, mean serum levels of POPs increase with age due to their lipophilicity and thus may have a greater effect in the elderly as opposed to early or middle age.^33^

Chronic hyperglycemia has been demonstrated to increase AGE‐RAGE signaling by promoting downstream outcomes such as fibroblast differentiation, measured by α‐SMA, RAGE expression, and signaling proteins of oxidative stressors as observed in the current study. These fibroblast‐mediated outcomes have been shown to exacerbate extracellular remodeling to decrease cardiac function.^30^, ^34^, ^35^ While the exact mechanisms governing POPs‐induced cardiac remodeling remain unknown, our current data indicate that RAGE and α‐SMA protein expression were significantly increased in the presence of POPs exposure, particularly in WT‐POPs mice, above that of WT mice. These observations are the first of its kind to suggest a possible role for POPs as a promoter of the AGE‐RAGE signaling cascade and subsequent cardiac remodeling. Since POPs exposure has been positively correlated with hyperglycemia and T2DM, and T2DM complications, including cardiac remodeling as indicated by left ventricular function and/or hypertrophy as described in previous studies, we aimed to determine the role of POPs exposure and the potential mechanisms responsible for increased incidence of maladaptive responses resulting from AGE‐RAGE signaling.^7^, ^10^, ^36^

The AGE‐RAGE signaling cascade activation has been shown to increase proinflammatory and oxidative stress modulators, such as NF‐κB, SOD‐1, and SOD‐2, to adapt to changes in inflammation and ROS levels.^19^, ^26^ NF‐κB protein expression was shown to be significantly diminished in *ob*/*ob*‐POPs mice. Decreases in NF‐κB levels oftentimes coincide with loss of protective mechanisms against apoptosis.^37^ This finding may indicate an inability of the *ob*/*ob*‐POPs animals to sustain a sufficient inflammatory response to POPs exposure. Further studies would need to be performed in order to confirm significance of these findings. In the current study, protein expression levels of two key neutralizers of ROS, SOD‐1 and SOD‐2, were measured to determine the compensatory ability of animals to adapt to POPs as potential increased oxidative stressors.^24^ It was observed that POPs exposure significantly reduced both SOD‐1 and ‐2 levels in *ob*/*ob*‐POPs hearts. No significant differences were observed between *ob*/ob and WT‐POPs mice as compared to WT. Singularly, POPs exposure or T2DM, may not alter SOD‐1 or SOD‐2 protein expression. These results suggest the combinatorial effect of POPs exposure and elevated AGE‐RAGE signaling would reduce innate compensatory mechanisms to inadequately protect against oxidative stressors. Alternatively, POPs‐generated and T2DM‐mediated oxidative stressors may be compensated for by SOD‐1 and SOD‐2 protein levels; however, in *ob*/*ob‐*POPs mice reduced SOD‐1 and SOD‐2 may not be able to offset rising ROS levels due to POPs exposure. Histological evaluation of H&E slides showed no differences in neutrophil invasion into the myocardium particularly in POPs treated groups. While prior studies have used neutrophil population as a predictor for changes in SOD levels, our findings suggest changes in neutrophil numbers may have either occurred early in POPs exposure timeframe or depleted over time with chronic exposure culminating in decreased SOD levels.^38^, ^39^ Future studies will be performed using known positive control agents, such as doxorubicin. Furthermore, additional studies will need to be performed in order to understand the exact mechanism.

In states of metabolic dysfunction such as obesity and diabetes, ROS levels become exceedingly high, and when compounded with POPs exposure, innate antioxidant defense mechanisms become overwhelmed with increased levels of ROS from the activation of RAGE/NF‐κB signaling.^26^, ^32^ Thus, decreased SOD expression in diabetic‐POPs treated animals may have dampened the cardioprotective mechanisms needed to offset POPs‐mediated oxidative and inflammatory stressors. Studies focused on the effects on fish oil contaminated with POPs on inflammation and oxidative stress and the effects of POPS exposure on hepatic gene expression profiles both noted a decrease in SOD enzyme activities in the presence of POPs contamination.^10^ Previously published results are consistent with our results and suggest future studies are needed to investigate molecular interactions between POPs in the inhibition of antioxidant activity. Therefore, exposure to the most prevalent environmental POPs could act as causative agents to promote cardiovascular remodeling through the AGE‐RAGE cascade.

Ventricular fibrosis has been demonstrated to be a significant outcome of increased RAGE activation and myofibroblast differentiation.^30^ We found both WT‐POPs and *ob*/*ob*‐POPs mouse hearts had significantly decreased soluble and insoluble collagen levels as well as decreased soluble and insoluble collagen levels, and consequently soluble‐insoluble ratios were also significantly decreased in the aforementioned groups. Changes in soluble levels are indicative of changes in collagen production and/or collagen degradation by MMPs.^29^, ^30^ While changes in insoluble collagen represent alterations in collagen maturation and crosslinking leaving collagen strands susceptible to degradative enzymes.^40^ Both, of which, can directly impact cardiac performance.^29^, ^30^, ^40^ Similar findings were also observed in picric acid‐sirius red stained ventricular cross‐sections (Figure 1); however, the percent of stained collagen was only significantly decreased in *ob*/*ob*‐POPs hearts. Without cardiac functional analysis, it is difficult to speculate on significance of findings. One implication of these findings may be attributed to the loss of NF‐κB transcriptional regulation affects collagen synthesis.^41^, ^42^, ^43^ Alternatively, loss of SOD‐1 and ‐2 in *ob*/*ob*‐POPs could result in a potential elevation in ROS levels resulting in elevated MMP activity, and consequently, increased collagen degradation.^44^, ^45^, ^46^ Of note, collagen levels were not significantly elevated in *ob*/*ob* hearts, which leptin has been noted to play a role in fibrosis.^47^, ^48^, ^49^

In summary, this study implicated organic pollutants (POPs) as potential promoters of RAGE protein expression. Our findings demonstrate that exposure to POPs may increase cardiometabolic risk and cardiac remodeling, due in part to POPs‐induced activation of the AGE‐RAGE signaling cascade. Results also showed that the POPs‐induced decreases in NF‐κB, SOD‐1, and SOD‐2 to potentially dampen cardioprotective mechanisms needed to offset POPs‐mediated oxidative and inflammatory stressors in the *ob*/*ob*‐POPs mice. The mechanisms by which this occurs is currently not understood. Therefore, the combination of these results with previous studies highlight the critical role of AGE‐RAGE cascade activation in the pathogenesis of T2DM and implicate this pathway in environmental perturbation of the cardiac ramifications of T2DM. The positive correlation between POPs exposure and onset of T2DM give rise to the need for future studies analyzing the potential mechanisms connecting POPs exposure to increased RAGE expression and subsequent adverse cardiac outcomes.

## AUTHOR CONTRIBUTIONS

All authors contributed equally to this article.
